# Proteomic analysis of urinary extracellular vesicles highlights specific signatures for patients with primary aldosteronism

**DOI:** 10.3389/fendo.2023.1096441

**Published:** 2023-05-08

**Authors:** Lorenzo Bertolone, Annalisa Castagna, Marcello Manfredi, Domenica De Santis, Francesca Ambrosani, Elisa Antinori, Paolo Mulatero, Elisa Danese, Emilio Marengo, Elettra Barberis, Mariangela Veneri, Nicola Martinelli, Simonetta Friso, Francesca Pizzolo, Oliviero Olivieri

**Affiliations:** ^1^ Department of Medicine, Unit of Internal Medicine, University of Verona, Verona, Italy; ^2^ Department of Translational Medicine, University of Piemonte Orientale, Novara, Italy; ^3^ Center for Translational Research on Autoimmune and Allergic Diseases, University of Piemonte Orientale, Novara, Italy; ^4^ Department of Medical Sciences, Division of Internal Medicine and Hypertension University of Torino, Torino, Italy; ^5^ Section of Clinical Biochemistry, University and Azienda Ospedaliera Universitaria Integrata of Verona, Verona, Italy; ^6^ Department of Sciences and Technological Innovation, University of Piemonte Orientale, Alessandria, Italy

**Keywords:** urinary extracellular vesicles (UEVs), primary aldosteronism, essential hypertension, proteomics, aquaporin 1 (AQP1), aquaporin 2 (AQP2), α-1-acid glycoprotein (AGP1)

## Abstract

**Background:**

Urinary extracellular vesicles (uEVs) can be released by different cell types facing the urogenital tract and are involved in cellular trafficking, differentiation and survival. UEVs can be easily detected in urine and provide pathophysiological information “*in vivo*” without the need of a biopsy. Based on these premises, we hypothesized that uEVs proteomic profile may serve as a valuable tool in the differential characterization between Essential Hypertension (EH) and primary aldosteronism (PA).

**Methods:**

Patients with essential hypertension (EH) and PA were enrolled in the study (EH= 12, PA=24: 11 Bilateral Primary Aldosteronism subtype (BPA) and 13 Aldosterone Producing Adenoma (APA)). Clinical and biochemical parameters were available for all the subjects. UEVs were isolated from urine by ultracentrifugation and analysed by Transmission Electron Microscopy (TEM) and nanotrack particle analysis (NTA). UEVs protein content was investigated through an untargeted MS-based approach. Statistical and network analysis was performed to identify potential candidates for the identification and classification of PA.

**Results:**

MS analysis provided more than 300 protein identifications. Exosomal markers CD9 and CD63 were detected in all samples. Several molecules characterizing EH *vs* PA patients as well as BPA and APA subtypes were identified after statistical elaboration and filtering of the results. In particular, some key proteins involved in water reabsorption mechanisms, such as AQP1 and AQP2, were among the best candidates for discriminating EH *vs* PA, as well as A1AG1 (AGP1).

**Conclusion:**

Through this proteomic approach, we identified uEVs molecular indicators that can improve PA characterization and help in the gain of insights of the pathophysiological features of this disease. In particular, PA was characterized by a reduction of AQP1 and AQP2 expression as compared with EH.

## 1 Introduction

Hypertension is a complex disease and a major cardiovascular risk factor, representing a leading cause of mortality and morbidity worldwide (1). Essential or primary hypertension (EH) accounts for nearly 90% of cases, while secondary hypertension, where a specific cause of high BP can be identified, represents the remaining 10%. Primary aldosteronism (PA) is the major form of secondary hypertension and is often associated with resistant hypertension. It is caused by a renin-independent increase in circulating aldosterone, which in most cases, can be ascribed to two major subtypes of adrenal tissue hyperproliferation: an unilateral aldosterone excess, mainly due by an aldosterone producing adenoma (APA) or a bilateral primary aldosteronism (BPA) caused by a two-sided aldosterone excess. The two subtypes drastically differ also in terms of preferential treatment, being the surgical removal of the adenoma the preferential choice for APA while the pharmacological intervention with Mineralcorticoid Receptor Antagonists (MRAs) is the usual option for BPA. The molecular mechanisms of PA have been partially uncovered in recent years, but much work is still needed to improve our understanding of the intrinsic and extrinsic pathways, especially related to the lack of response to BP-lowering drugs (2). Overall, the current work-up for the diagnosis of PA is cumbersome, time-consuming, relatively expensive, and it requires specific technical skills or experience, thus limiting its potential routine application on the multitude of the hypertensive population. Considering the high prevalence of PA among hypertensive patients, the increased risk of PA subjects in developing cardiovascular events and the availability of specific treatments that can control or even heal the disease, an effective diagnostic work- up is extremely valuable.

Urine extracellular vesicles (UEVs) have gained significant interest in the recent years as a suitable source of potential useful markers for the study of many diseases and as a valuable tool in diagnosis and health state monitoring (3, 4). They represent a heterogeneous population of vesicles originating from several sections of the urogenital tract, including kidneys and bladder, and from residing immune cells, yeast and bacteria (3). UEVs contain a cargo composed mainly of proteins, nucleic acids, lipids etc. and are involved in several processes of renal communication, including proximal-to-distal signaling, developmental regulation, control of ion transport, regulation of inflammation, immune response and even elimination of cellular waste (4).

Their isolation is non-invasive and, since their cargo is protected by a lipid bilayer, and it theoretically reflects the pathophysiological condition of the cell-of-origin, low-abundant proteins and RNA that are contained in uEVs can be good putative indicators (5, 6). For these reasons, several studies have explored the possibility of using uEVs as a source of molecules for the diagnosis and characterization of several diseases including kidney injury, Chronic Kidney Disease (CKD), Acute Kidney Injury (AKI), glomerular injury, kidney fibrosis, cancer, diabetes mellitus and infections (7, 8).

Exosomes can be valuable also for a deeper investigation of PA and hypertension features: few studies have shown that uEVs cargo of patients suffering of mineralocorticoid-dependent arterial hypertension are enriched in proteins and miRNA related to salt reabsorption activity and regulation (9–11) and recently Alpha-1-Acidic Glycoprotein (A1AG1 or AGP1), found in UEVs, was proposed as potential biomarker of PA (12). The recent efforts in the standardization, isolation protocols, the creation of dedicated databases and tools for uEVs have considerably improved the research in the field, though many critical aspects still remain to be fully addressed (3, 13).

For the study of complex traits such as hypertension, omics approaches offer the advantage of a global molecular picture as they allow the identification of all factors involved in the pathophysiological mechanisms of the disease (14). The application of proteomics to study hypertension diseases is particularly promising also in the frame of risk stratification and diagnosis (15).

The main aim of our study was thus to analyze the proteomic profile of uEVs from EH and PA patients and to obtain information on specific molecular signatures potentially useful for classification and characterization of this disease.

## 2 Materials and methods

### 2.1 Hypertensive subjects

Patients referring to two different Hypertension Units (Verona and Turin, Italy) were enrolled between 2010 and 2015, including patients with urine samples sufficient for exosomes extraction. Selection criteria were applied to avoid confounding factors: no antihypertensive drug other than verapamil and/or alpha-blockers were allowed during the previous 4 weeks and hypokalemia was corrected, if present. Hypertensive women receiving oral contraceptive therapy were excluded. The criteria for PA diagnosis were as previously reported (16, 17). In brief, orthostatic ARR (aldosterone-to-renin ratio) higher than the reference value established for each laboratory (32 pg/ml for ratio of aldosterone, expressed as pg/ml, to direct active renin, expressed as pg/ml (18); 40 for ratio of aldosterone expressed as ng/dl to renin activity expressed as ng/ml•h in Turin (17)) and positive iv Salt Loading Test (SLT). The test was positive for PAC levels higher than 50 pg/ml (17). In the case of PA diagnosis, the classification for subtypes (i.e. BPA or APA) was based on both adrenal CT and AVS (19). For this study 11 BPA and 13 APA subjects (total of PA patients=24) were evaluated. Other hypertensive subjects, diagnosed as EH (n= 12), were included in the study, having orthostatic ARR lower than the reference value for PA screening.

The study was conducted according to the principles contained in the Declaration of Helsinki, each patient gave written informed consent, and the protocol was approved by the Institutional Review Board Ethical Committee of our institution.

### 2.2 Sample collection

The second morning urine samples were collected for each subject, as well as blood samples for biochemical routine parameters analysis. Urine samples were collected and processed according to a previously reported protocol (20). Briefly, the second morning urine samples were collected, chilled on ice and processed within one hour from collection. Urine pH was adjusted to 7.0 and a protease inhibitor cocktail was added (Complete Protease Inhibitor; Roche Diagnostics, Basel, Switzerland) before centrifugation at 3500 rpm for 40 minutes at 4°C. Supernatant was subsequently filtered using 0.22 µm filters (Millipore, Billerica, Massachusetts, USA). Cell-free urine (CFU) was then stored in aliquots at -20°C. Urine processed as illustrated here were then used for further analysis. Measurements of biochemical and hormonal parameters were performed by the Laboratory of the Verona and Turin University Clinical Chemistry Institutes as previously described (17, 18).

### 2.3 UEVs isolation and proteins extraction

Ultracentrifugation protocol was adapted from Pisitkun et al. with adjustments suggested by Livshits and colleagues (21). Aliquots of urine (10 ml) were centrifuged at 17000 g for 15 minutes at 4°C to remove urinary sediment, including whole cells, large membrane fragments and other debris. Supernatant was then centrifuged at 100000 g for 120 minutes at 4°C to obtain a low-density membrane pellet. This pellet was resuspended in 400 µl of ammonium bicarbonate 100 mM (AMBIC).

### 2.4 TEM analysis

UEVs pellet was thawed and resuspended in 100 µl of sterile PBS and kept at +4°C. Aliquots of 6 µl of the suspension were absorbed for 1 minute on an ultra-thin carbon coated copper grid (CF200H-Cu-UL, Electon Microscopy Sciences) and excess of suspension was removed by gentle blotting. Suspension adsorbed to grid was placed on 1 drop of UranyLess solution (Electon Microscopy Sciences) for 1 second. Operation was repeated and the second drop was left in place 30 seconds. Grid was then dried by gentle blotting and air. Sample was then visualized on a Morgagni 268D (FEI Philips) transmission electron microscope, setting the voltage to 80kV.

### 2.5 Nanoparticles tracking analysis

Particle size distribution in urine and uEVs samples were determined using NanoSight NS300 system (Malvern Technologies, Malvern, UK). Light scattering and Brownian motion are used to determine particles size and distribution of small particles suspended in solution (20-2000nm). Particles’ movement is observed through a microscope and their size is calculated using Stokes-Einstein equation. All the samples were diluted 1:250 with physiological saline. Instrument settings were selected according to the manufacturer’s software manual. Samples were analyzed under constant flow conditions (flow rate = 20) at 25°C according to manufacturer recommendations. Three videos of 60s were captured with camera level of 14/15. The data were analyzed using instrument’s software with a detection threshold of 5/6.

### 2.6 Mass spectrometry analysis

Sample processing for MS analysis and data collection were conducted at the Mass Spectrometry unit of the University of Piemonte Orientale (Novara, Italy). Proteins extracted from uEVs were quantified using BCA assay (Pierce BCA protein assay kit; ThermoFisher Scientific). Samples were denaturated with TFE, reduced in DTT 200 mM and alkylated with IAM 200 mM before complete tryptic digestion with 2 μg of Trypsin/Lys-C (Promega, Madison, WI, USA). Digested peptides were desalted on the Discovery^®^ DSC-18 solid phase extraction (SPE) 96-well Plate (25 mg/well) (Sigma-Aldrich Inc., St. Louis, MO, USA) and vacuum evaporated to be reconstituted with 20 μL of 0.05% formic acid in water.

Trypsin-digested sample proteins were analyzed with a micro-LC Eksigent Technologies (Eksigent Technologies, Dublin, CA, USA) system that included a micro LC200 Eksigent pump with flow module 5-50 µL, interfaced with a 5600+ TripleTOF system (Sciex, Concord, ON, Canada) equipped with DuoSpray Ion Source and CDS (Calibrant Delivery System). The stationary phase was a Halo C18 column (0.5 x 100 mm, 2.7 µm; Eksigent Technologies, Dublin, CA, USA). The mobile phase was a mixture of 0.1% (v/v) formic acid in water (A) and 0.1% (v/v) formic acid in acetonitrile (B), eluting at a flowrate of 15.0 µL min−1 at an increasing concentration of solvent B from 2% to 40% in 30 min. For identification purposes, the samples were subjected to a data dependent acquisition (DDA): the mass spectrometer analysis was performed using a mass range of 100–1500 Da (TOF scan with an accumulation time of 0.25 s), followed by a MS/MS product ion scan from 200 to 1250 Da (accumulation time of 5.0 ms) with the abundance threshold set at 30 cps (35 candidate ions can be monitored during every cycle).

For the label-free quantification the samples were subjected to cyclic data independent analysis (DIA) of the mass spectra, using a 25-Da window: the mass spectrometer was operated such that a 50-ms survey scan (TOF-MS) was performed and subsequent MS/MS experiments were performed on all precursors. These MS/MS experiments were performed in a cyclic manner using an accumulation time of 40 ms per 25-Da swath (36 swaths in total) for a total cycle time of 1.5408 s. The ions were fragmented for each MS/MS experiment in the collision cell using the rolling collision energy. The MS data were acquired with Analyst TF 1.7 (Sciex, Concord, ON, Canada). Two DDA and three DIA acquisitions were performed. The DDA files were searched using Protein Pilot software v. 4.2 (Sciex, Concord, ON, Canada) and Mascot v. 2.4 (Matrix Science Inc., Boston, MA, USA). The UniProt Swiss-Prot reviewed database containing human proteins (version 01/02/2018, containing 42271 sequence entries) was used and a target-decoy database search was performed. False Discovery Rate was set at 1%. 

### 2.7 Statistical analysis

Shapiro-Wilk test was used to test variables for normality (**Table 1**). Frequencies were analysed through Chi-square test. Univariate statistical analysis was performed using GraphPad Prism (v. 9.4.1), R (v. 3.6.1) and SPSS (v. 20). Hiearchical clustering, ROC curves and combined ROC curves were built using MetaboAnalyst (v 5.0).

**Table 1:** Biochemical and hormonal features of patients affected by Primary Aldosteronism (PA) and Essential Hypertension (EH).

	EH patients (n=12)	PA patients (n=24; 13 APA, 11 BPA)	P-value
Sex (M;F)	8;4	15;9	0.81
Age (years)	40.15 (1.4)	49.1 (1.194)	0.39
BMI (kg/m^2^)	27.33 ± 2.645	28.2 ± 4.032	0.57
Renin (pg/ml)	13.7 (1.84)	1.8 (4.876)	<0.05
Aldosterone (pg/ml)	141 (1.635)	335.3 (1.67)	<0.01
ARR	10.64 (1.946)	77.82 (2.107)	<0.01
Serum K (mmol/l)	3.921 (1.09)	3.316 (1.23)	<0.01
Glucose (mmol/l)	4.92 ± 0.49	5.1 ± 0.23	0.57
Cholesterol (mg/dl)	188.7 ± 33.224	185.3 ± 16.65	0.87
Triglycerides (mg/dl)	79.83 (1.67)	102.2 (1.41)	0.28
U-Creatinine (mg/dl)	210 ± 74.19	102.8 ± 89.43	<0.01
U-Sodium (mmol/l)	127.4 ± 66.7	64.05 ± 37.6	<0.01
U-Potassium (mmol/l)	45.01 (2.26)	31.14 (2.17)	0.24
U-Chloride (mmol/l)	105.4 (2.24)	55.99 (2.19)	<0.05

For normally distributed variables, values are indicated as mean ± SD and T-test p-value is reported. Geometric means, geometric SD factor and Mann-Whitney p-value are reported otherwise.

## 3 Results

We analysed the proteomic content of uEVs extracted from urine of PA and EH patients, through an untargeted MS approach. The biochemical and clinical characteristics of the patients with EH or PA diagnosis are reported in **Table 1**. As expected, aldosterone, renin, K and ARR were significantly different in PA patients in comparison to EH.

**Figure 1:**
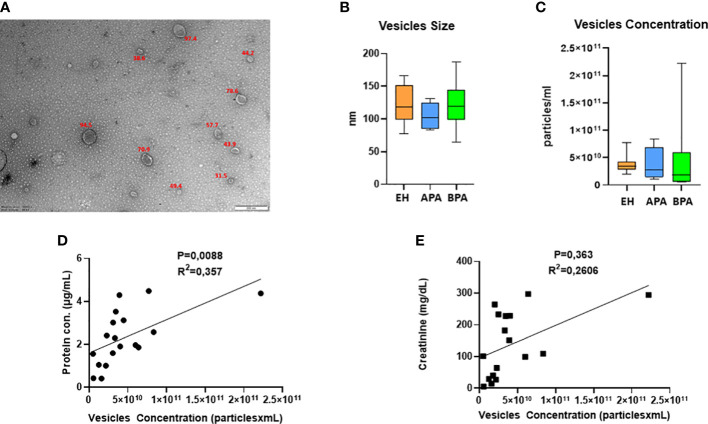
UEVs characterization: **(A)** representative picture of TEM characterization of uEVs isolated with ultracentrifugation; **(B, C)** NTA data on size **(B, C)** concentration of uEVs, error bar shows the range. UEVs concentration in relation to urinary creatinine **(D)** and protein content of the isolated vesicles **(E)**.

The isolated uEVs were characterized by TEM and NTA, that confirmed an average vesicle size compatible to what is reported in literature for exosomes (**Figures 1A, B**). Across the different groups of patients, uEVs did not significantly differ in terms of concentration and size (**Figures 1B, C**). Moreover, uEVs concentration correlated with protein content measured by BCA but not with urinary creatinine (**Figures 1D, E**). Total protein load was applied as normalization factor for MS analysis. Untargeted MS analysis identified a total of 301 proteins. A complete list of the identifications and raw data are available in the supplementary materials (**Supplementary Table 1**). Univariate T-test and fold-change analysis were initially used to shortlist proteins differentially abundant across the experimental groups. Exosomal and urinary exosomal markers such as CD9, CD63 and AQP2 were detected by MS in all the samples.

### 3.1 Proteomic analysis of PA *vs* EH

We initially compared the uEVs proteome of EH *vs* PA patients to identify proteins that can help in the identification of PA in a population of hypertensive subjects.

**Figure 2:**
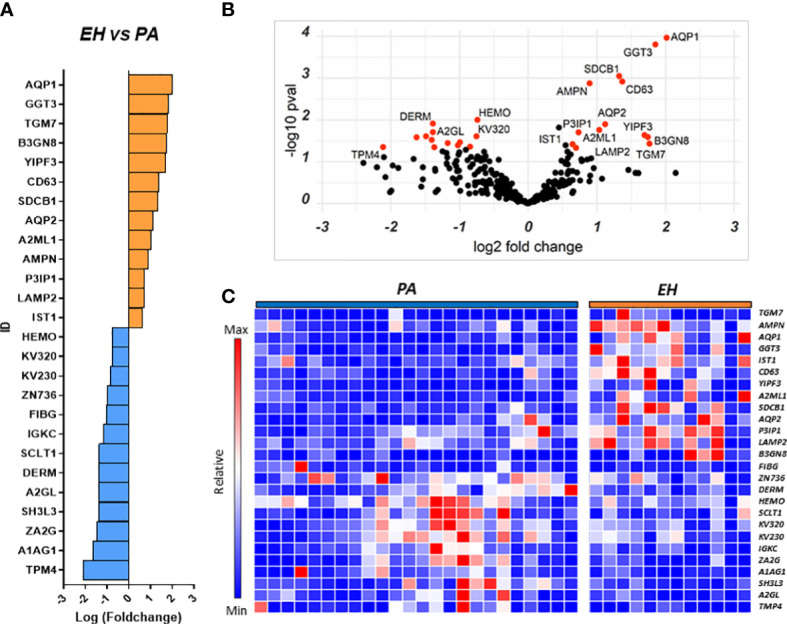
Proteins regulated in the EH vs PA comparison. Proteins resulting from the comparison EH *vs* PA are represented in: **(A)** Barplot of Log(fc) of statistically significant proteins (Pval < 0.05) with a fold change cutoff of 1.5. Proteins more abundant in EH are indicated as orange bars and proteins more abundant in PA as blue bars. **(B)** Volcano plot and distribution of P values and fold change of the positive hits; **(C)** Heatmap of the top 26 proteins resulting from t-test analysis. Each protein is indicated with its abbreviated gene name alias.

**Figure 3:**
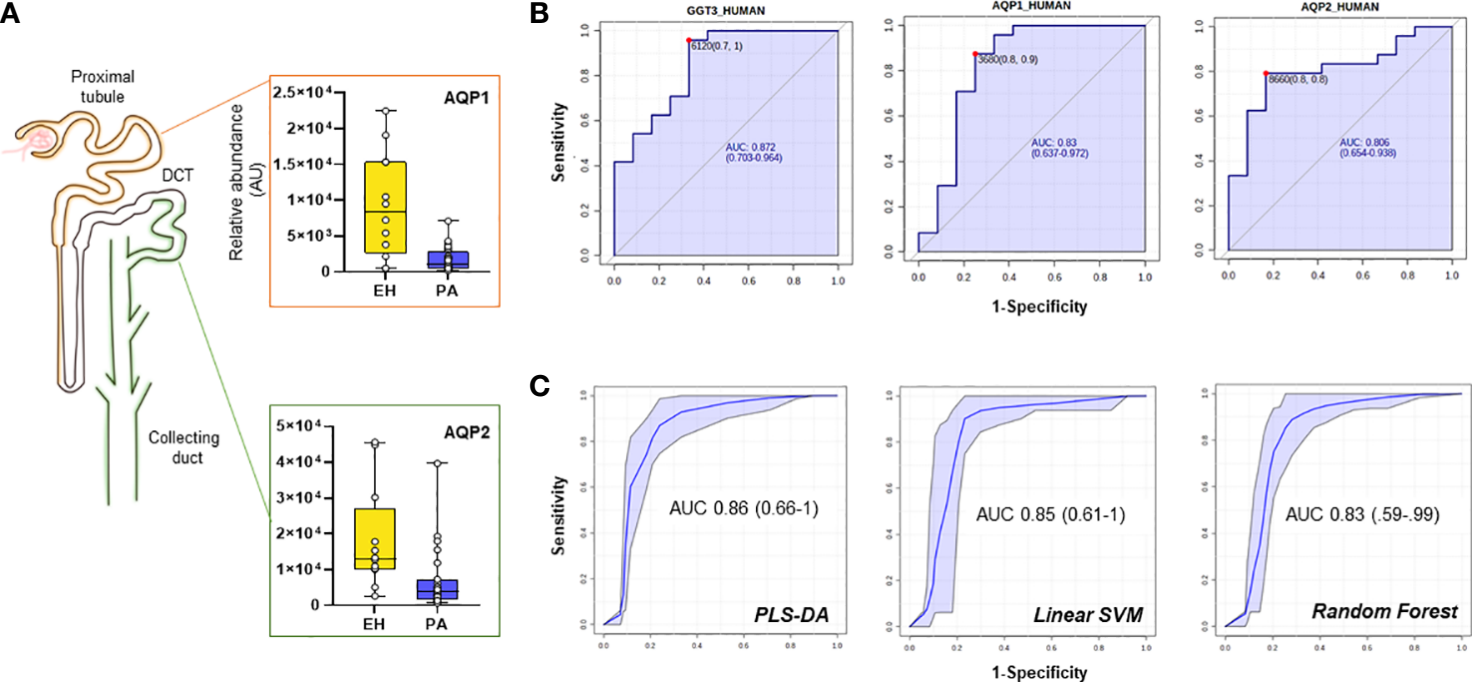
Candidate biomarkers for the EH *vs* PA comparison. **(A)** Boxplots and relative position in the nephron of AQP1, AQP2 **(B)** ROC curves of the best hits in the comparison according to AUC and **(C)** target Multi ROC combining the best 6 selected features (AUC≥0.80).

Several proteins (n=26) were differentially regulated between the two conditions, 13 of which were upregulated in PA (**Figures 2A–C**). To assess the performance of the putative candidates, we applied Receiver Operating Characteristic analysis (ROC) on the proteins significantly different in EH and PA. We found six proteins that appear to be able to differentiate EH from PA patients (top 3 ROC curves are shown in **Figure 3B**) The following molecules had the best diagnostic performances: Putative glutathione hydrolase 3 proenzyme (AUC=0.87), Aminopeptidase N (AUC=0.83), CD63 antigen (AUC=0.83), Aquaporin-1 (AUC=0.83), IST1 homolog (AUC=0.82) and Aquaporin-2 (AUC=0.81) (**Figures 3A, B**). The combined ROC curves of these six best proteins performed with PLS-DA, linear SVM and Random Forest reported an AUCs of 0.86, 0.85 and 0.83 respectively (**Figure 3C**).

A combined ROC was also constructed for AQP1 and AQP2 only, since they both share a key role in determining the final urine volume, but are positionally separated along the renal tubulus. Optimized threshold (**Figure 3B**) for the two aquaporins was used to calculate confusion matrixes of the single markers and of the combination of the two. Screening performance of the combination of the two markers was compared using PPV (Positive Predictive Value) and NPV (Negative Predictive Value). The combination of the two markers slightly improved the AUC with respect to the markers alone. By combining the conditions in which at least one of the two AQP values was above the optimized threshold, NPV values reached 0.92 with a PPV of 0.79 (**Supplementary Table 3**).

Subsequently, network analysis was attempted to highlight potential pathways involved in the disease and in the different regulations of uEVs proteins using Gene Ontology and the database STRING (https://string-db.org/) focusing on protein-protein interactions and functional networks (**Supplementary Figure 1**). All the linked proteins found to be higher in EH patients were associated to exosomes biogenesis by the STRING node analysis. One of such protein was CD63, a member of the tetraspanins family. CD63 was found in association with LAMP2 (lysosomal associated membrane protein-2), a glycoprotein frequently found in exosomes and late endosomes: significant correlations were found with other proteins more abundant in EH patients involved in exosomes biogenesis (SDCB1) and members of the ESCRTIII complex (IST1) (CD63-SDCB1: Spearman r=0.55, p-value=<0.001; CD63-IST1: r= 0.46, p-value=0.005). While the CD63+ fraction of uEVs was more abundant in EH patients, we detected no significant differences in the abundance of CD9, another known marker of exosomes and extracellular vesicles (**Supplementary Figure 1**). Furthermore, both AQP1 and AQP2 were found to significantly correlate with CD63 marker (Spearman r=0.32 and r=0.64 respectively) but only AQP2 was found in correlation with CD9 (r=0.56, all p-values<0.05).

A subset of proteins significantly altered between PA and EH in our setting have been already proposed as urinary biomarkers in other studies or have been observed to be altered under specific conditions. The list of such proteins is reported in **Supplementary Table 2** along with a brief description of their functions and some information on the specific studies in which they were found.

### 3.2 Proteomic analysis of APA *vs* BPA

**Figure 4:**
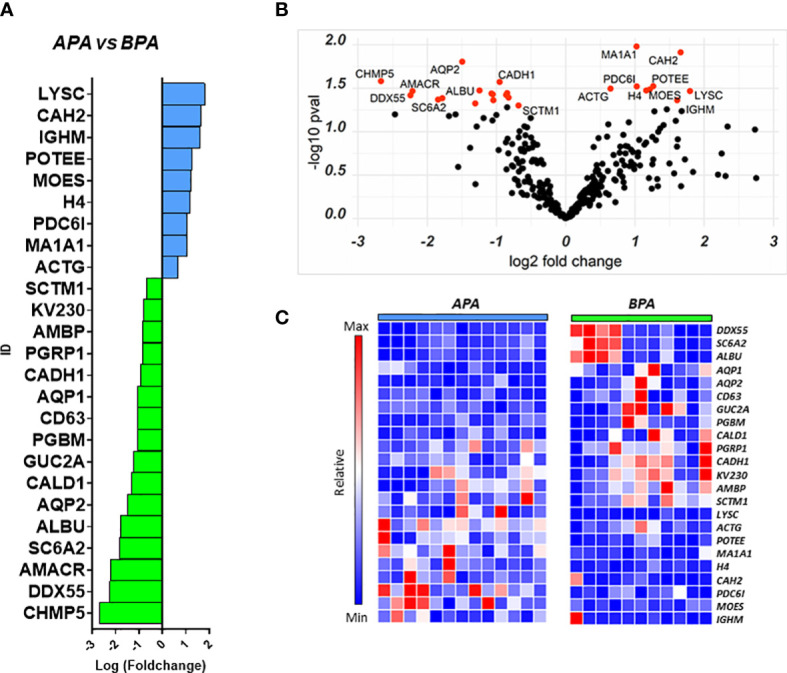
Proteins regulated in the APA vs BPA comparison. Proteins resulting from the comparison between the 2 PA subtypes are represented in: **(A)** Barplot of Log(fc) for proteins with Pval < 0.05 and FC cutoff of 1.5. Proteins more abundant in APA are indicated as blue bars and proteins more abundant in BPA are indicated asgreen bars; **(B)** Volcano plot and distribution of P values and fold change of the positive hits; **(C)** Heatmap of the top 25 proteins resulting from t-test analysis. Each protein is indicated with its abbreviated gene name alias.

We performed a similar analysis to identify proteins that could separate the two main PA subgroups and help in the characterization of the disease. We compared the uEVs proteome of 13 APA patients against 11 BPA patients finding a total of 25 differentially regulated proteins. Nine proteins were more abundant in APA while 16 were higher in BPA patients (**Figures 4A–C**).

**Figure 5:**
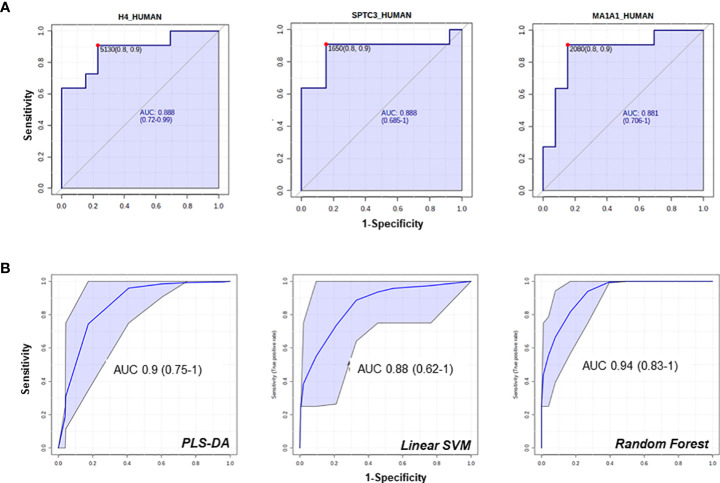
ROC curves of candidate biomarkers for the comparison APA vs BPA. ROC curves of the best hits in the comparison between the 2 APA subtypes **(A, B)** multi ROC curves obtained combining the best 6 selected features (AUC≥0.84).

Among the proteins with different abundance in BPA and APA, BPA group had a higher level of CD63, and again AQP1, AQP2 that were suggested as candidate biomarkers for differentiating PA vs EH patients in the previous comparison. In this case, while univariate analysis revealed significant differences, none of these targets reached the arbitrary threshold we set for AUC and CI in the shortlisting of the best candidates (AUC>0.8, lower CI>0.6). The statistical analysis also showed that APA patients can be discriminated from the BPA ones using the following markers: Histone H4 (AUC=0.89), Serine palmitoyltransferase 3 (SPTC3, AUC=0.89), Mannosyl-oligosaccharide 1,2-alpha-mannosidase IA (MA1A1, AUC=0.89), Lysozyme C (LYSC, AUC=0.85), Heat shock protein beta-1 (HSPB1, AUC=0.84) and Immunoglobulin lambda variable 3-25 (IGLV3-25, AUC=0.85) (**Figure 5A**). By performing the combined ROC curve of these six best proteins we obtained an AUC of 0.91, 0.89 and 0.94 for PLS-DA, linear SVM and Random Forest, respectively (**Figure 5B**).

To detect functional protein associations, network analysis was also carried out on the proteins resulting from fold-change analysis. GO analysis for biological process identified several proteins involved in transport processes (*vesicle-mediated transport*, *renal water transport*, *bicarbonate transport*). Proteins involved in water transport and vesicle-mediated transport were up-regulated in BPA patients while structural proteins (ACT1, MSN) were up-regulated in APA. Although STRING identified several protein-protein interactions, no relevant nodes were found, since interacting proteins were differentially regulated between the two experimental groups (**Supplementary Figure 2**).

## 4 Discussion

In our study we aimed to identify putative protein candidates in uEVs that can help in the identification and characterization of PA. Fold-change analysis and ROC analysis identified six possible candidates that can help in the identification of PA patients in a population of hypertensive subjects. In the list of the top candidates, we report the presence of two members of the family of Aquaporins (AQP1 and 2) (22) more abundant in EH-derived UEVs. Aquaporins are membrane proteins that are regulated by several stimuli and allow the osmotic passage of water molecules across the cell membrane. Taking into account the notable amount of volume filtered daily by the kidney, aquaporins expressed at the level of the renal tubule are therefore crucial in water homeostasis (22). PA is a condition typically characterized by an elevated and persistent water and sodium retention as compared with EH, due to the aldosterone excess. The present results indicate a reduced abundance of membrane AQPs in PA patients when compared to EH patients, an effect more marked for the APA group.

Interestingly AQP2 and AQP1 belong to different districts and are also regulated by different and independent pathways, suggesting a broad “systemic” response. AQP1 is localized in the apical and basolateral membrane of the epithelial cell in the proximal tubule, and it is stimulated by ANGII that directly affects the expression of AQP1 mRNA (23). Differences in the abundance of AQP1 between PA and EH (5.5-fold in EH over PA) may be at least partially explainable by the status of the RAAS in PA patients, in whom plasma aldosterone concentration is high, and renin is suppressed. Conversely, AQP2 is located in the apical plasma membrane in the collecting duct (CD), part of what is known as aldosterone-sensitive nephron, and it is the primary target for AVP regulation. This regulation acts on the trafficking of AQP2-loaded vesicles to the apical region or by increasing AQP2 mRNA expression (24). Although it has been reported that aldosterone may have some effects on AQP2 down-regulation (25, 26), we hypothesize that the reduced abundance of AQP2 in PA patients may be part of a broader response to limit the contribution of passive water transportation, in face of an abnormal unrestrained reabsorption driven by aldosterone. Another possible explanation for this result is an alteration in vesicle trafficking and release, that may be AVP-directed. Previous reports have suggested that PA patients have a higher copeptin level, the C-terminal portion of provasopressin (27). Although in our setting AVP levels were not available we think that the assessment of the vasopressin signaling axis in PA could give useful information and is worthy of further investigations.

Vesicles parameters characterization (size and concentration) did not provide notable differences between the experimental groups investigated. MS analysis showed the presence of many acknowledged markers of uEVs such as CD9, CD63 and AQP2 as our isolation method was not based on a specific immunological selection of vesicles but allowed for a broad heterogeneous presence of different uEVs populations.

It is possible to speculate that the profile of surface markers and the identification of tissue-specific cargo inside the vesicles could give useful information on the preferred source/localization in the nephron of the vesicles originating cells. Correlation analysis in fact showed that AQP2 was positively correlated with vesicles concentration while CD9 and CD63 did not correlate, indicating that a large fraction of vesicles derived from the CD section of the nephron and bore AQP2.

AQP2 is also commonly known as a marker of urinary extracellular vesicles and may also reflect differences in the abundance of vesicles originating from the CD. AQP1 and AQP2 protein levels in exosomes has been previously shown to directly correlate with their renal protein level (with some exceptions (28)), and it has been observed that the urinary fraction of AQP2 is mainly located in exosomes with preserved transport activity (29, 30), supporting the observations that uEVs content can be used to non-invasively extract valuable information on the pathophysiology of the nephron.

Another known uEVs marker is the member of the family of tetraspanins CD63 which, together with CD9, it is considered to be a reliable surface marker for exosomes and extracellular vesicles. CD63 has been found to be more abundant in uEVs isolated from EH patients (the lowest levels have been measured in APA patients), while no difference in abundance was detected for CD9. Previous studies have shown that surface markers of EV can be associated with specific cargos and that CD63 influences water reabsorption by altering the trafficking of vesicles associated with transporters (31, 32). We have found that CD63 is in positive correlation with other proteins involved in vesicles biogenesis and, indeed, both AQP1 and AQP2. Although the clinical significance of the observed differences in CD63 remains unclear, they are likely reflective of the heterogeneity of the vesicles population across the patient groups, especially considering that uEVs were not significantly different in quantity or size.

Among the proteins resulting from the fold-change analysis of the EH vs PA comparison, we have found also A1AG1 (AGP1), a protein that is recently attracting attention as a potential biomarker for PA (12, 33). A1AG1 is an acute-phase protein associated with inflammation, it has been suggested to indicate increased cardiovascular risk, and its upregulation in uEVs of PA patients may be a consequence of mineralocorticoid activation (12). Our results further consolidate a possible role as PA indicator for this protein in urine, taking into consideration also previously found associations with other conditions, including chronic heart failure or bladder cancer (see **Supplementary Table 2**).

Other uEVs known markers of tubular origin might also be differently associated to EH and PA and to uEVs. In particular, Neutrophil Gelatinase-Associated Lipocain (NGAL), a quite recent and emergent molecule involved in acute kidney injury (AKI) and measured in uEVs, could be potentially evaluated (34). NGAL was recently observed to be involved in MR induced cardiovascular damage, but its role in PA pathophysiology is still not clear, thus we plan to evaluate it in further studies (35).

Viable alternatives that allow to separate the two most representative subtypes of PA (APA and BPA) other than the current standard procedure (AVS) are strongly desirable. Here we present a list of markers that can be considered possible candidates to help in this task. ROC analysis suggested that particularly histone H4, SPTC3, MA1A1, LYSC, HSPB1 and IGLV3 can effectively discriminate the two PA subtypes we considered. However, another interesting protein that was not included in the 6-best candidates ROC shortlist is CAH2 (**Figure 4A**). This enzyme is significantly more abundant in APA-derived uEVs and is expressed along the nephron, interacting with a large number of transporters and ion exchangers, including the epithelial sodium-proton exchanger NHE3, pendrin and AQP1 (36, 37). Considering the central role of CAH2 in regulating water reabsorption and the statistically significant differences in AQP1 between APA and BPA, we believe it worth further consideration.

Multiparametric ROC analysis is a valuable instrument that combines several signals to obtain models with improved sensitivity and specificity. In our specific setting we could not find significant improvements in the parameters of combined ROC curves compared to the performance of the single-component ones to differentiate PA vs EH. However, an improvement was identified in the combined ROC curves for the APA vs BPA comparison, especially using PLS-DA and Random Forest algorithms. The combination of predictive power of several markers is a strategy that can improve sensitivity in the characterization of PA. In fact, as regards AQP1 and 2, the application of a combined analysis of these proteins, may be a viable solution to increase the NPV of the classification.

In previous studies, we demonstrated striking metabolic differences in urine samples of the two PA subtypes, and in the present uEVs proteomic study, we detected differences in water reabsorption proteins and key regulators of acid-base equilibrium and ion transporters. Our exploratory study on uEVs proteomics has pioneered the possibility to exploit differences in vesicles cargo and composition for improving in a non-invasive way the identification and characterization of PA subjects. We acknowledge that a limitation of this study is the number of enrolled patients that did not allow for in-depth stratification, identification of sex-specific biomarker candidates. Moreover, our experimental workflow does not rely on the latest LC technology for MS, which could have provided a higher number of protein IDs. Nevertheless, it is interesting to note that our data independently confirm and extend the observation that A1AG1 is a valid candidate biomarker for PA and that they are in general suggestive of differences in the water reabsorption regulation between EH and PA and between PA subtypes. The rationale of our analysis was the characterization of the uEVs proteome of hypertensive patients with PA, using samples collected at the enrolment. A prospective advancement may be aimed at investigating the effects of PA treatment (either surgical removal of the adrenal or medical treatment with MR antagonist) on the uEVs proteome, to confirm the relevance for the disease of the proteins selected in this study.

In conclusion, the results of the study offer two important insights for future research. The first could be of clinical and practical utility, while the second may have theoretical and pathogenetic relevance.

Indeed, if confirmed, the results indicate that quantifying urinary expression of AQPs-in addition to Aldosterone and ARR-may help in the diagnostic workup (confirming or excluding the diagnosis of PA in 9 out of 10 patients). The second suggestion from the present results is that the system regulating water homeostasis during PA is dysregulated with possible dissociation between levels of AVP hormone and its receptors (ie. AQPs) even if further experimental verification is needed to confirm this hypothesis.

## Data availability statement

The original contributions presented in the study are included in the article/**Supplementary Material**. Further inquiries can be directed to the corresponding author.

## Ethics statement

The studies involving human participants were reviewed and approved by Azienda Ospedaliera Universitaria Integrata. The patients/participants provided their written informed consent to participate in this study.

## Author contributions

“Conceptualization, LB, AC, FP and OO. Validation, NM, EB, EM. Investigation, FA, EA, DD and LB. Resources, FP and PM. Data curation, MV and ED. Writing—original draft preparation, AC. Writing—review and editing, LB, PM, FP, SF. Visualization, MM, NM and EB. Supervision, OO and EM. Funding acquisition, FP, SF, OO. All authors contributed to the article and approved the submitted version.
