# Supplementary material for: Proteomic analysis of urinary extracellular vesicles highlights specific signatures for patients with primary aldosteronism
Source: Front Endocrinol (Lausanne). 2023 May 8;14:1096441. doi: 10.3389/fendo.2023.1096441 (PMC10200877; doi:10.3389/fendo.2023.1096441)
Supplement: Supplementary file 1 [file Presentation_1.pptx]

## Slide 1
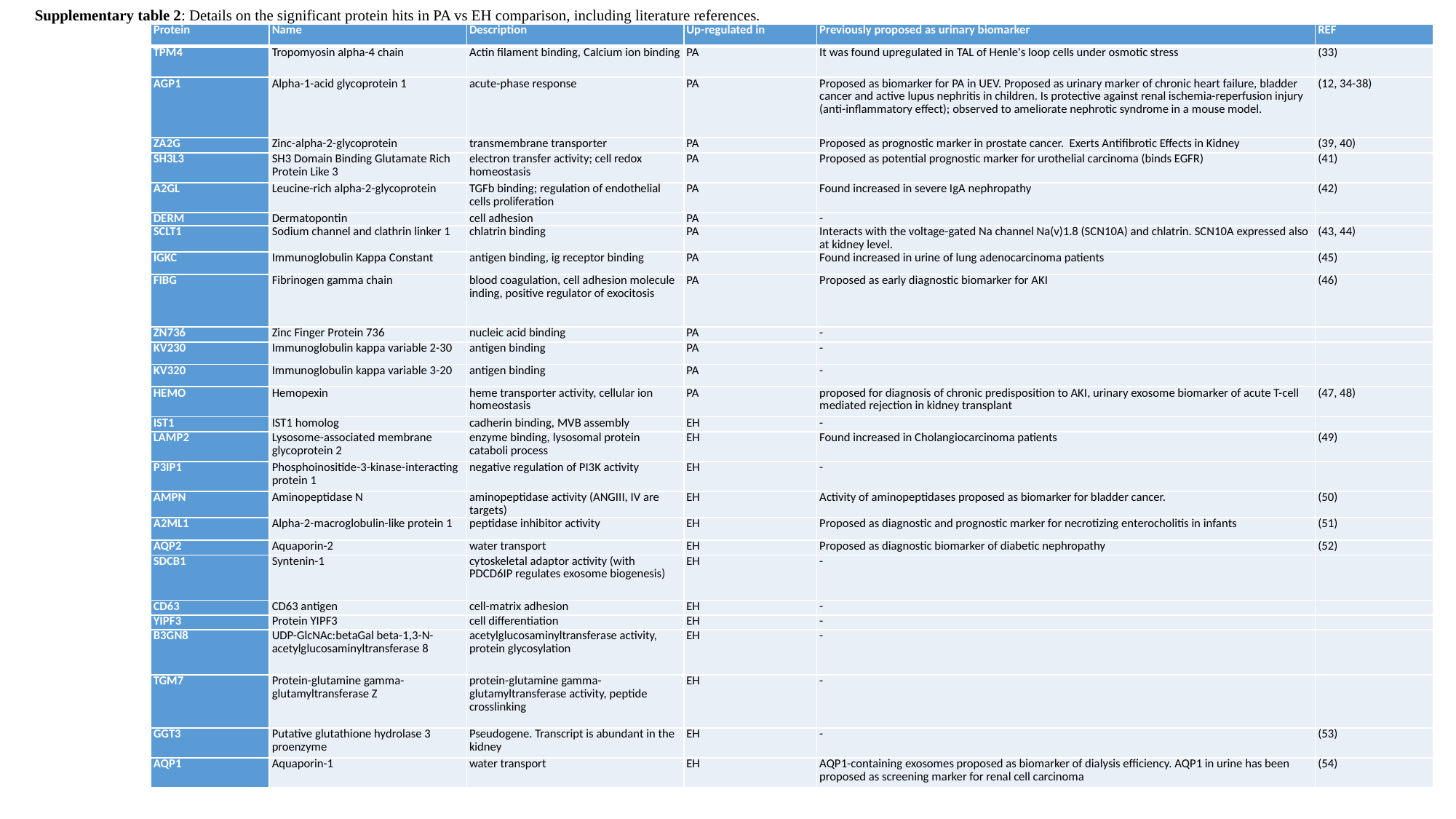

Supplementary table 2: Details on the significant protein hits in PA vs EH comparison, including literature references.
| Protein | Name | Description | Up-regulated in | Previously proposed as urinary biomarker | REF |
| --- | --- | --- | --- | --- | --- |
| TPM4 | Tropomyosin alpha-4 chain | Actin filament binding, Calcium ion binding | PA | It was found upregulated in TAL of Henle's loop cells under osmotic stress | (33) |
| AGP1 | Alpha-1-acid glycoprotein 1 | acute-phase response | PA | Proposed as biomarker for PA in UEV. Proposed as urinary marker of chronic heart failure, bladder cancer and active lupus nephritis in children. Is protective against renal ischemia-reperfusion injury (anti-inflammatory effect); observed to ameliorate nephrotic syndrome in a mouse model. | (12, 34-38) |
| ZA2G | Zinc-alpha-2-glycoprotein | transmembrane transporter | PA | Proposed as prognostic marker in prostate cancer. Exerts Antifibrotic Effects in Kidney | (39, 40) |
| SH3L3 | SH3 Domain Binding Glutamate Rich Protein Like 3 | electron transfer activity; cell redox homeostasis | PA | Proposed as potential prognostic marker for urothelial carcinoma (binds EGFR) | (41) |
| A2GL | Leucine-rich alpha-2-glycoprotein | TGFb binding; regulation of endothelial cells proliferation | PA | Found increased in severe IgA nephropathy | (42) |
| DERM | Dermatopontin | cell adhesion | PA | - | |
| SCLT1 | Sodium channel and clathrin linker 1 | chlatrin binding | PA | Interacts with the voltage-gated Na channel Na(v)1.8 (SCN10A) and chlatrin. SCN10A expressed also at kidney level. | (43, 44) |
| IGKC | Immunoglobulin Kappa Constant | antigen binding, ig receptor binding | PA | Found increased in urine of lung adenocarcinoma patients | (45) |
| FIBG | Fibrinogen gamma chain | blood coagulation, cell adhesion molecule inding, positive regulator of exocitosis | PA | Proposed as early diagnostic biomarker for AKI | (46) |
| ZN736 | Zinc Finger Protein 736 | nucleic acid binding | PA | - | |
| KV230 | Immunoglobulin kappa variable 2-30 | antigen binding | PA | - | |
| KV320 | Immunoglobulin kappa variable 3-20 | antigen binding | PA | - | |
| HEMO | Hemopexin | heme transporter activity, cellular ion homeostasis | PA | proposed for diagnosis of chronic predisposition to AKI, urinary exosome biomarker of acute T-cell mediated rejection in kidney transplant | (47, 48) |
| IST1 | IST1 homolog | cadherin binding, MVB assembly | EH | - | |
| LAMP2 | Lysosome-associated membrane glycoprotein 2 | enzyme binding, lysosomal protein cataboli process | EH | Found increased in Cholangiocarcinoma patients | (49) |
| P3IP1 | Phosphoinositide-3-kinase-interacting protein 1 | negative regulation of PI3K activity | EH | - | |
| AMPN | Aminopeptidase N | aminopeptidase activity (ANGIII, IV are targets) | EH | Activity of aminopeptidases proposed as biomarker for bladder cancer. | (50) |
| A2ML1 | Alpha-2-macroglobulin-like protein 1 | peptidase inhibitor activity | EH | Proposed as diagnostic and prognostic marker for necrotizing enterocholitis in infants | (51) |
| AQP2 | Aquaporin-2 | water transport | EH | Proposed as diagnostic biomarker of diabetic nephropathy | (52) |
| SDCB1 | Syntenin-1 | cytoskeletal adaptor activity (with PDCD6IP regulates exosome biogenesis) | EH | - | |
| CD63 | CD63 antigen | cell-matrix adhesion | EH | - | |
| YIPF3 | Protein YIPF3 | cell differentiation | EH | - | |
| B3GN8 | UDP-GlcNAc:betaGal beta-1,3-N-acetylglucosaminyltransferase 8 | acetylglucosaminyltransferase activity, protein glycosylation | EH | - | |
| TGM7 | Protein-glutamine gamma-glutamyltransferase Z | protein-glutamine gamma-glutamyltransferase activity, peptide crosslinking | EH | - | |
| GGT3 | Putative glutathione hydrolase 3 proenzyme | Pseudogene. Transcript is abundant in the kidney | EH | - | (53) |
| AQP1 | Aquaporin-1 | water transport | EH | AQP1-containing exosomes proposed as biomarker of dialysis efficiency. AQP1 in urine has been proposed as screening marker for renal cell carcinoma | (54) |

## Slide 2
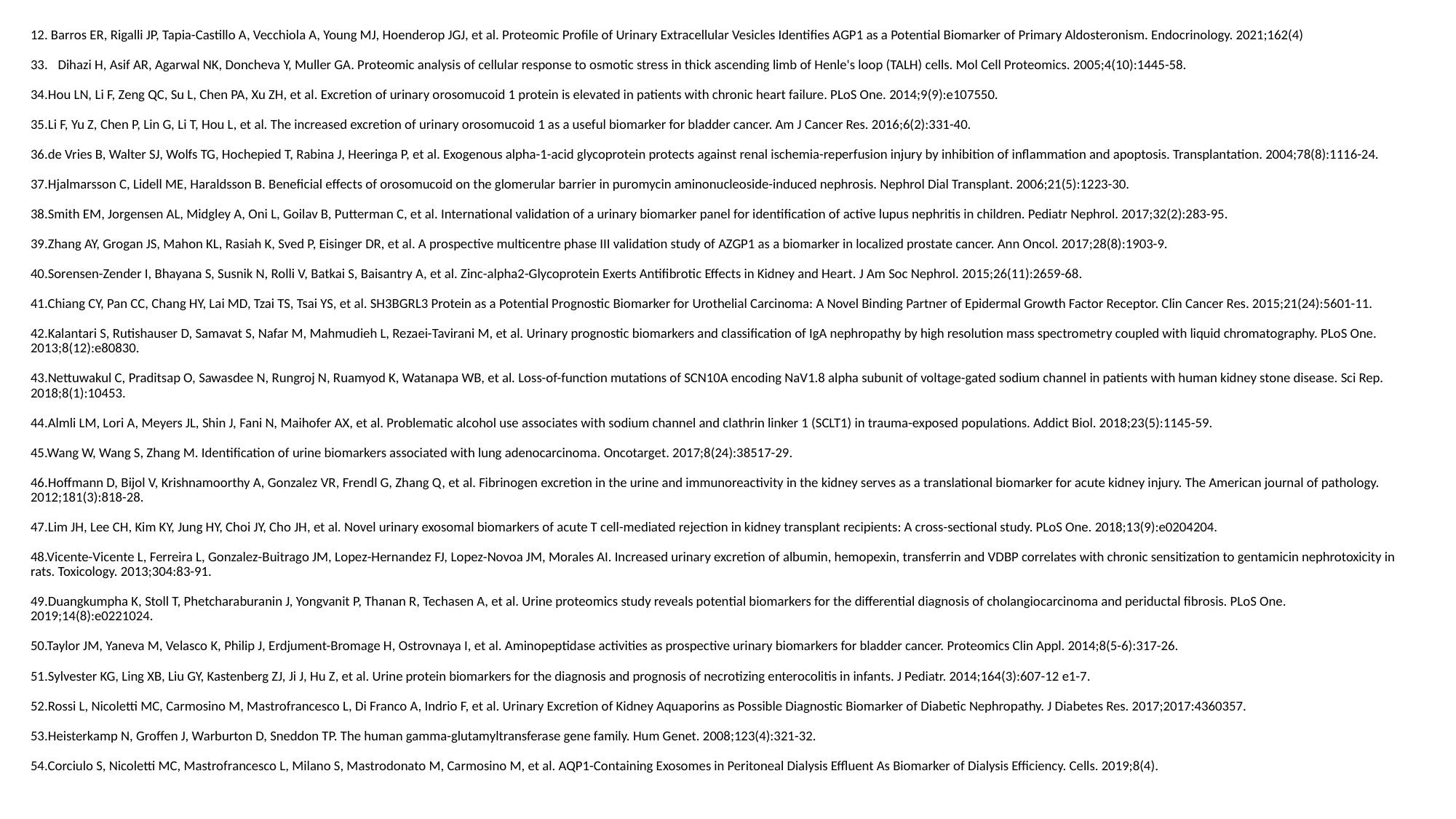

12. Barros ER, Rigalli JP, Tapia-Castillo A, Vecchiola A, Young MJ, Hoenderop JGJ, et al. Proteomic Profile of Urinary Extracellular Vesicles Identifies AGP1 as a Potential Biomarker of Primary Aldosteronism. Endocrinology. 2021;162(4)
Dihazi H, Asif AR, Agarwal NK, Doncheva Y, Muller GA. Proteomic analysis of cellular response to osmotic stress in thick ascending limb of Henle's loop (TALH) cells. Mol Cell Proteomics. 2005;4(10):1445-58.
34.Hou LN, Li F, Zeng QC, Su L, Chen PA, Xu ZH, et al. Excretion of urinary orosomucoid 1 protein is elevated in patients with chronic heart failure. PLoS One. 2014;9(9):e107550.
35.Li F, Yu Z, Chen P, Lin G, Li T, Hou L, et al. The increased excretion of urinary orosomucoid 1 as a useful biomarker for bladder cancer. Am J Cancer Res. 2016;6(2):331-40.
36.de Vries B, Walter SJ, Wolfs TG, Hochepied T, Rabina J, Heeringa P, et al. Exogenous alpha-1-acid glycoprotein protects against renal ischemia-reperfusion injury by inhibition of inflammation and apoptosis. Transplantation. 2004;78(8):1116-24.
37.Hjalmarsson C, Lidell ME, Haraldsson B. Beneficial effects of orosomucoid on the glomerular barrier in puromycin aminonucleoside-induced nephrosis. Nephrol Dial Transplant. 2006;21(5):1223-30.
38.Smith EM, Jorgensen AL, Midgley A, Oni L, Goilav B, Putterman C, et al. International validation of a urinary biomarker panel for identification of active lupus nephritis in children. Pediatr Nephrol. 2017;32(2):283-95.
39.Zhang AY, Grogan JS, Mahon KL, Rasiah K, Sved P, Eisinger DR, et al. A prospective multicentre phase III validation study of AZGP1 as a biomarker in localized prostate cancer. Ann Oncol. 2017;28(8):1903-9.
40.Sorensen-Zender I, Bhayana S, Susnik N, Rolli V, Batkai S, Baisantry A, et al. Zinc-alpha2-Glycoprotein Exerts Antifibrotic Effects in Kidney and Heart. J Am Soc Nephrol. 2015;26(11):2659-68.
41.Chiang CY, Pan CC, Chang HY, Lai MD, Tzai TS, Tsai YS, et al. SH3BGRL3 Protein as a Potential Prognostic Biomarker for Urothelial Carcinoma: A Novel Binding Partner of Epidermal Growth Factor Receptor. Clin Cancer Res. 2015;21(24):5601-11.
42.Kalantari S, Rutishauser D, Samavat S, Nafar M, Mahmudieh L, Rezaei-Tavirani M, et al. Urinary prognostic biomarkers and classification of IgA nephropathy by high resolution mass spectrometry coupled with liquid chromatography. PLoS One. 2013;8(12):e80830.
43.Nettuwakul C, Praditsap O, Sawasdee N, Rungroj N, Ruamyod K, Watanapa WB, et al. Loss-of-function mutations of SCN10A encoding NaV1.8 alpha subunit of voltage-gated sodium channel in patients with human kidney stone disease. Sci Rep. 2018;8(1):10453.
44.Almli LM, Lori A, Meyers JL, Shin J, Fani N, Maihofer AX, et al. Problematic alcohol use associates with sodium channel and clathrin linker 1 (SCLT1) in trauma-exposed populations. Addict Biol. 2018;23(5):1145-59.
45.Wang W, Wang S, Zhang M. Identification of urine biomarkers associated with lung adenocarcinoma. Oncotarget. 2017;8(24):38517-29.
46.Hoffmann D, Bijol V, Krishnamoorthy A, Gonzalez VR, Frendl G, Zhang Q, et al. Fibrinogen excretion in the urine and immunoreactivity in the kidney serves as a translational biomarker for acute kidney injury. The American journal of pathology. 2012;181(3):818-28.
47.Lim JH, Lee CH, Kim KY, Jung HY, Choi JY, Cho JH, et al. Novel urinary exosomal biomarkers of acute T cell-mediated rejection in kidney transplant recipients: A cross-sectional study. PLoS One. 2018;13(9):e0204204.
48.Vicente-Vicente L, Ferreira L, Gonzalez-Buitrago JM, Lopez-Hernandez FJ, Lopez-Novoa JM, Morales AI. Increased urinary excretion of albumin, hemopexin, transferrin and VDBP correlates with chronic sensitization to gentamicin nephrotoxicity in rats. Toxicology. 2013;304:83-91.
49.Duangkumpha K, Stoll T, Phetcharaburanin J, Yongvanit P, Thanan R, Techasen A, et al. Urine proteomics study reveals potential biomarkers for the differential diagnosis of cholangiocarcinoma and periductal fibrosis. PLoS One. 2019;14(8):e0221024.
50.Taylor JM, Yaneva M, Velasco K, Philip J, Erdjument-Bromage H, Ostrovnaya I, et al. Aminopeptidase activities as prospective urinary biomarkers for bladder cancer. Proteomics Clin Appl. 2014;8(5-6):317-26.
51.Sylvester KG, Ling XB, Liu GY, Kastenberg ZJ, Ji J, Hu Z, et al. Urine protein biomarkers for the diagnosis and prognosis of necrotizing enterocolitis in infants. J Pediatr. 2014;164(3):607-12 e1-7.
52.Rossi L, Nicoletti MC, Carmosino M, Mastrofrancesco L, Di Franco A, Indrio F, et al. Urinary Excretion of Kidney Aquaporins as Possible Diagnostic Biomarker of Diabetic Nephropathy. J Diabetes Res. 2017;2017:4360357.
53.Heisterkamp N, Groffen J, Warburton D, Sneddon TP. The human gamma-glutamyltransferase gene family. Hum Genet. 2008;123(4):321-32.
54.Corciulo S, Nicoletti MC, Mastrofrancesco L, Milano S, Mastrodonato M, Carmosino M, et al. AQP1-Containing Exosomes in Peritoneal Dialysis Effluent As Biomarker of Dialysis Efficiency. Cells. 2019;8(4).

## Slide 3
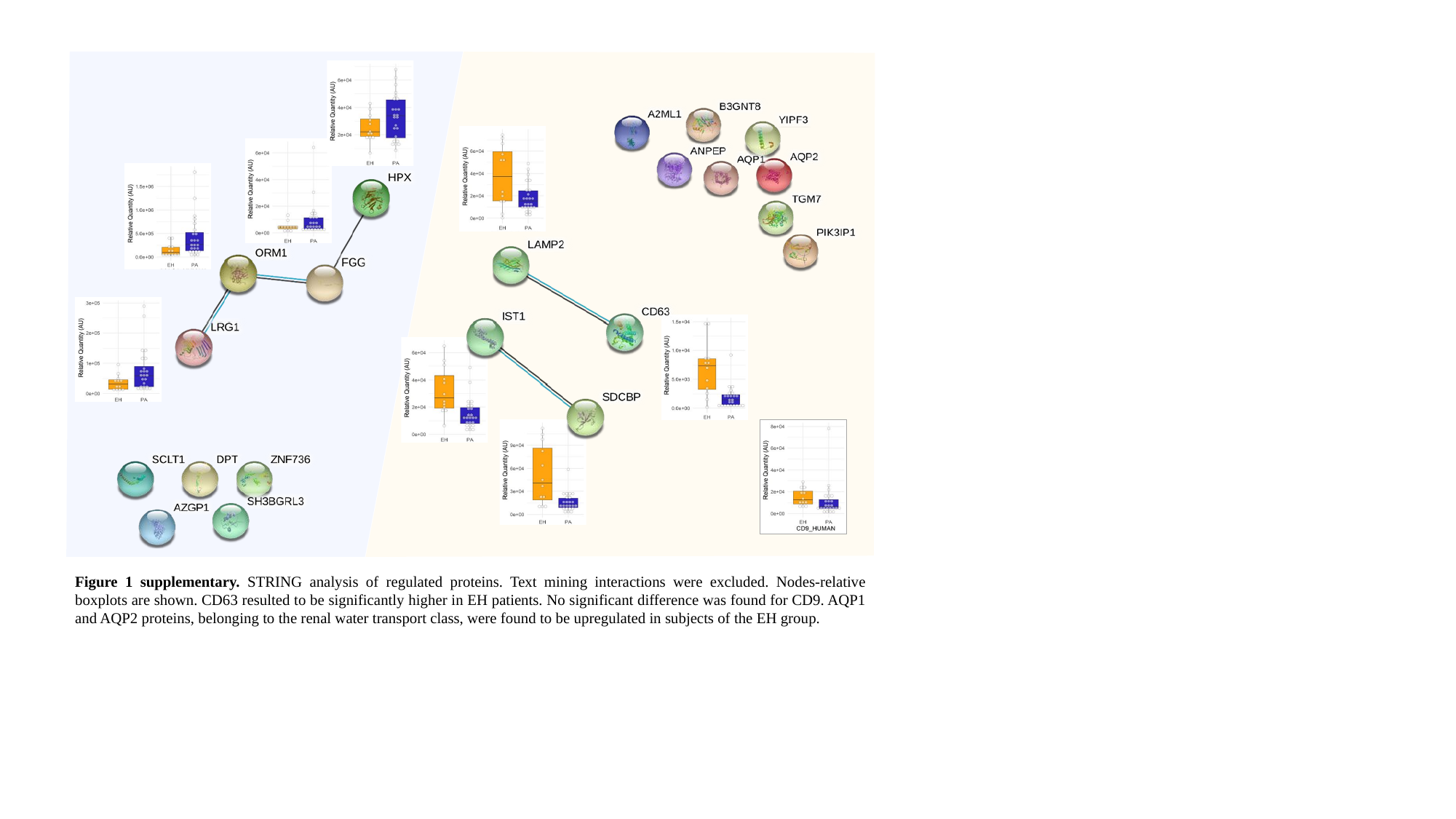

Figure 1 supplementary. STRING analysis of regulated proteins. Text mining interactions were excluded. Nodes-relative boxplots are shown. CD63 resulted to be significantly higher in EH patients. No significant difference was found for CD9. AQP1 and AQP2 proteins, belonging to the renal water transport class, were found to be upregulated in subjects of the EH group.

## Slide 4
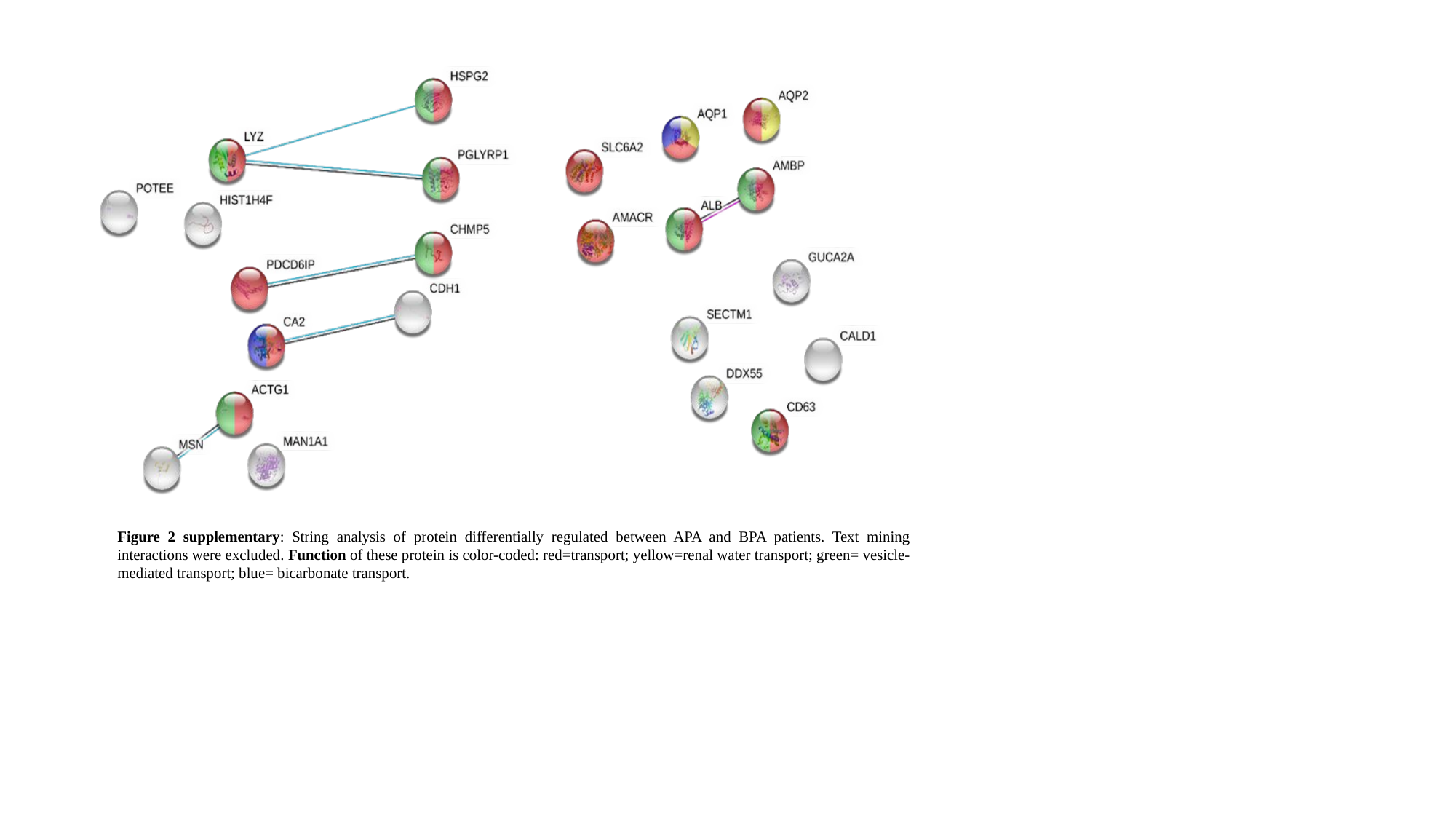

Figure 2 supplementary: String analysis of protein differentially regulated between APA and BPA patients. Text mining interactions were excluded. Function of these protein is color-coded: red=transport; yellow=renal water transport; green= vesicle-mediated transport; blue= bicarbonate transport.

## Slide 5
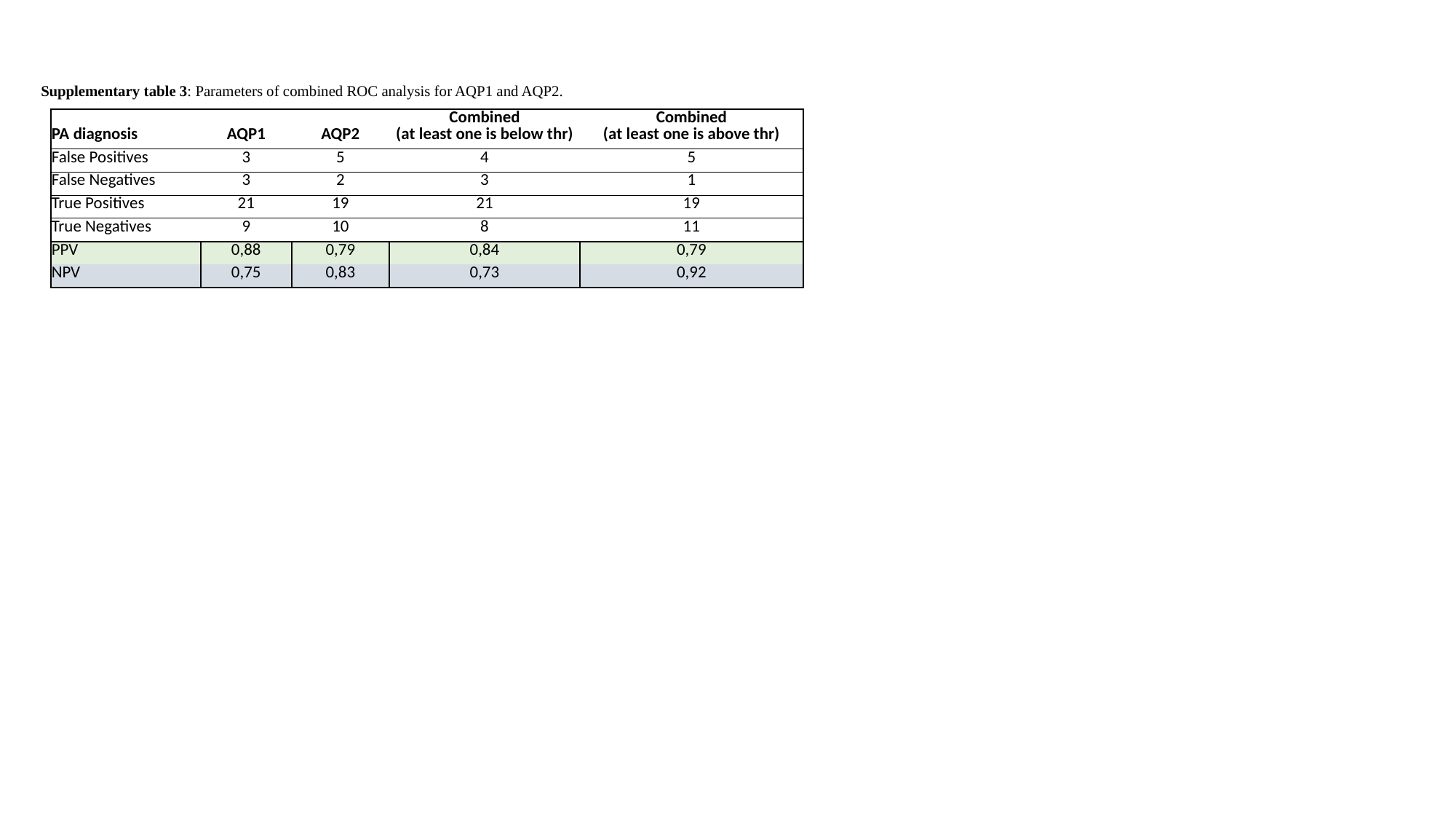

Supplementary table 3: Parameters of combined ROC analysis for AQP1 and AQP2.
| PA diagnosis | AQP1 | AQP2 | Combined (at least one is below thr) | Combined (at least one is above thr) |
| --- | --- | --- | --- | --- |
| False Positives | 3 | 5 | 4 | 5 |
| False Negatives | 3 | 2 | 3 | 1 |
| True Positives | 21 | 19 | 21 | 19 |
| True Negatives | 9 | 10 | 8 | 11 |
| PPV | 0,88 | 0,79 | 0,84 | 0,79 |
| NPV | 0,75 | 0,83 | 0,73 | 0,92 |
